# X-Linked Hypophosphatemic Rickets Manifesting as Sclerotic Bone Disease and Enthesopathy

**DOI:** 10.7759/cureus.10874

**Published:** 2020-10-10

**Authors:** Hiya Boro, Shailendra Singh Naik, Charandeep Singh, Saurav Khatiwada, Rajesh Khadgawat

**Affiliations:** 1 Endocrinology, All India Institute of Medical Sciences, New Delhi, IND; 2 Radiology, All India Institute of Medical Sciences, New Delhi, IND; 3 Endocrinology, Diabetes and Metabolism, All India Institute of Medical Sciences, New Delhi, IND

**Keywords:** rickets, enthesopathy, osteosclerosis, dental abscess, hypophosphatemia

## Abstract

X-linked hypophosphatemic (XLH) rickets is a genetic disease caused due to the inactivation of the PHEX gene (phosphate regulating gene with homology to endopeptidase on the X chromosome). The usual presentation is with rickets and osteomalacia, and dental abscesses leading to premature loss of teeth. However, enthesopathy and sclerotic bone disease in XLH have also been reported in a few case reports. In this report, we describe the case of a 23-year-old female patient who presented to us with severe bone deformities, proximal myopathy, truncal weakness, and recent onset of pain and stiffness around the joints. She was diagnosed with XLH and was found to have severe enthesopathy along with heterotopic ossification.

## Introduction

X-linked hypophosphatemic (XLH) rickets is a disease characterized by rachitic manifestations and osteomalacia in the form of bone pain, bone deformities, proximal myopathy, and poor dental health. It is caused by the inactivating mutation of the PHEX gene (phosphate regulating gene with homology to endopeptidase on the X chromosome). The PHEX gene encodes for an endopeptidase that causes cleavage of fibroblast growth factor 23 (FGF23), a phosphatonin that causes phosphate wastage from the body. In the presence of the PHEX gene mutation, FGF23 levels are significantly elevated, leading to phosphaturia and hypophosphatemia. Although XLH traditionally presents as a rachitic disorder [1], there have also been isolated reports of heterotopic ossification and enthesopathy [2-4]. The exact mechanism of enthesopathy is not known, but it may be due to the expansion of the mineralizing fibrocartilage cells [5]. The enthesopathy is usually resistant to conventional treatment with calcitriol and phosphate. Pain and stiffness may persist even after the resolution of other symptoms and the improvement of biochemical milieu.

## Case presentation

A 23-year-old female patient presented to us with progressive deformity of her lower limbs towards the right side. It had started at two years of age and had continued until 14 years. She had then undergone corrective surgery for windswept deformity, following which there had been no further progression. At 16 years of age, she had sustained bilateral hip fractures after trivial trauma. She had consulted an orthopedic surgeon for her fractures and had been advised surgery. However, the family had not complied with the advice, and the patient had restricted her treatment to the intake of calcium and vitamin D supplements. Subsequently, for the past seven years until her current presentation, she had been bed-bound. Gradually, she had developed contracture deformity of bilateral lower limbs.

The patient also had diffuse musculoskeletal pain that was moderate to severe in intensity. In the past six months, her pain had worsened. Also, in the past two years, she had developed gradually worsening low backache and stiffness. She had also developed stiffness around bilateral hip joints, elbows, and shoulders.

She had a history suggestive of proximal and truncal muscle weakness. She could not get up from her bed without support. Neurological examination revealed a power of 3/5 in the proximal muscles of both lower limbs and the truncal muscles. There was also a history of dental abscesses that had started at the age of five years. When she presented to us, she was edentulous.

She had received multiple intermittent courses of calcium, vitamin D, and calcitriol supplements without phosphate. She was the shortest among all her peers. Her exact height and weight could not be measured as she was bed-bound and had contracture deformity of lower limbs.

Her brother, who was 18 years old, also had a similar history of windswept deformity of lower limbs, along with proximal myopathy and dental abscesses. His complaints had also started at an early age of four years. The windswept deformity had progressed until the age of 16 years. His manifestations were not as severe as that of our patient. He had not had any fractures. He could also ambulate without support.

Investigations of the patient revealed hypophosphatemia [serum phosphate of 0.5 mmol/L (normal range: 0.8-1.5 mmol/L)] with low tubular maximum reabsorption of phosphate to glomerular filtration rate (TmP/GFR) [0.2 mmol/L (normal range: 0.9-1.4 mmol/L)], suggesting renal phosphate loss. On urine analysis, there was no aminoaciduria, bicarbonaturia, glucosuria, or low molecular weight proteinuria, unlike in the case of proximal renal tubular acidosis. Blood gas analysis also revealed a normal blood ph [ph: 7.41 (normal range: 7.35-7.45)] and normal serum bicarbonate levels [serum HCO_3_: 24 mmol/L (normal range: 24-28 mmol/L)]. She had a normal hemogram, liver, and kidney function tests. Her serum total calcium was 2.2 mmol/L (normal range: 2.1-2.6 mmol/L), serum 25-hydroxyvitamin D (25(OH)D) was 164.5 nmol/L (normal range: 75-250 nmol/l), while serum intact parathyroid hormone was 6.3 pmol/L (normal range: 1.5-6.8 pmol/L). Serum alkaline phosphatase (ALP) was markedly elevated at 1,154 IU/L (normal range: 80-240 IU/L), suggestive of active metabolic bone disease. Her serum FGF23 was elevated at 97 pg/ml (normal range in our laboratory: <35 pg/ml).

**Figure 1:**
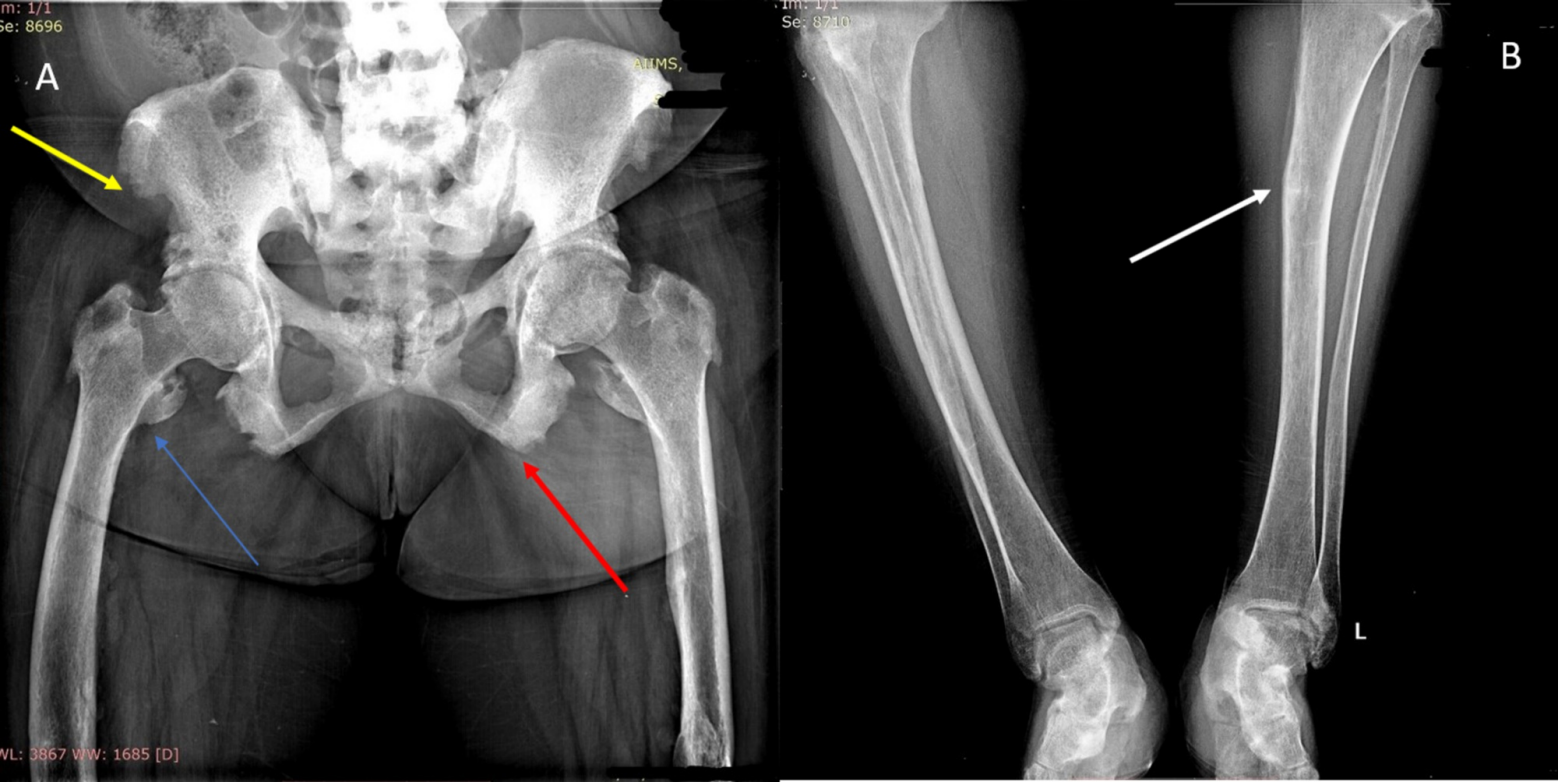
Image 1A showing enthesopathy and 1B showing osteopenia and insufficiency fracture 1A: pelvic radiograph anteroposterior view with proximal thighs showing irregularity at bilateral anterior superior iliac spine (yellow arrow), at the bilateral ischial tuberosity (red arrow), and at greater and lesser trochanters (blue arrow) suggestive of enthesopathy. Bilateral axial migration of femoral heads is present, suggesting protrusio acetabuli. 1B: bilateral leg radiograph showing osteopenic tibia and fibula along with insufficiency fracture at left tibial diaphysis (white arrow)

**Figure 2:**
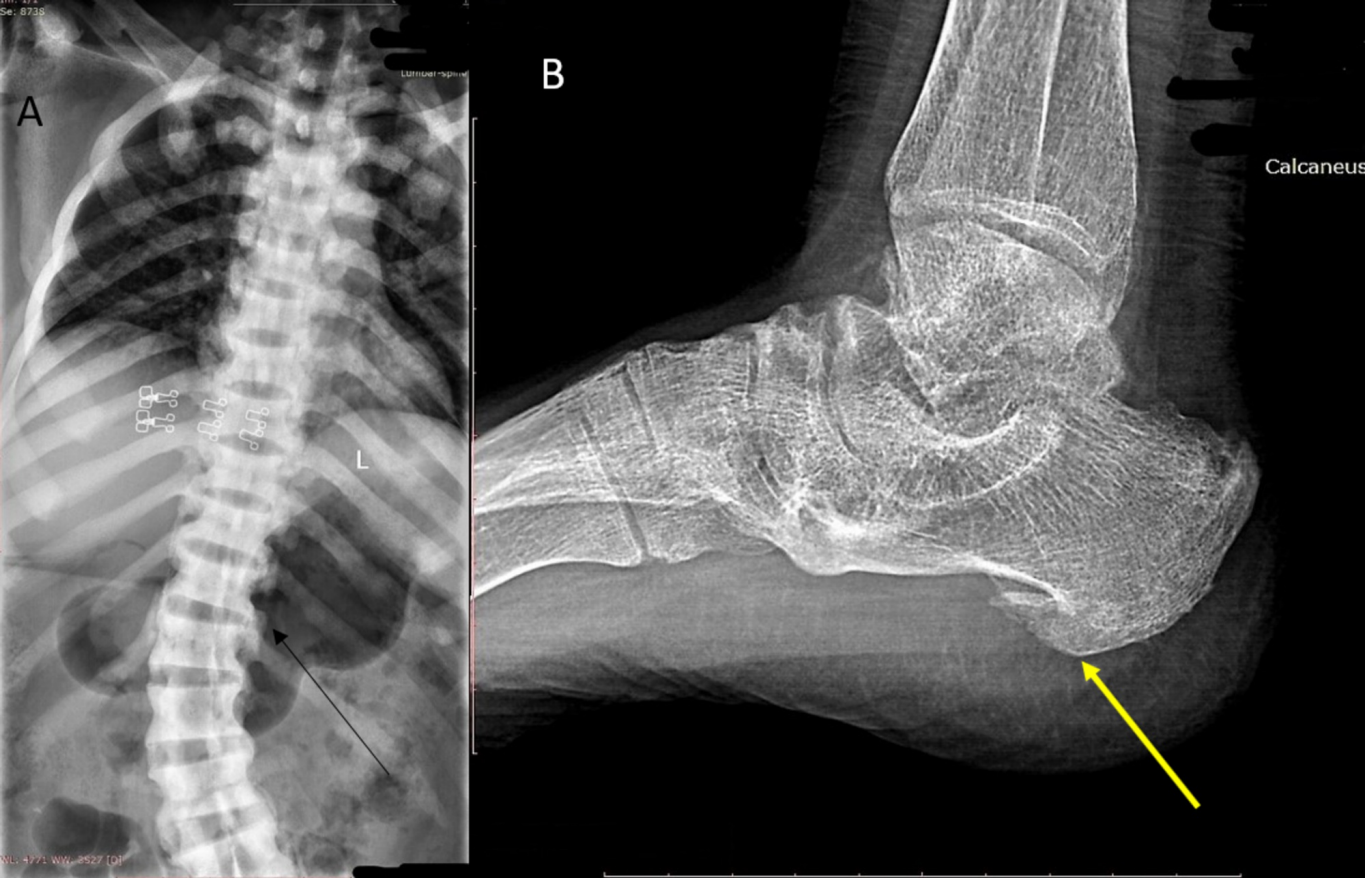
Image 2A showing osteophytic changes of spine and 2B showing calcaneal spurs 2A: dorsolumbar spine radiograph anteroposterior view showing bridging osteophytic changes and scoliotic deformity at lower dorsal vertebral level (black arrow). 2B: calcaneal spur representing degenerative change (yellow arrow)

**Figure 3:**
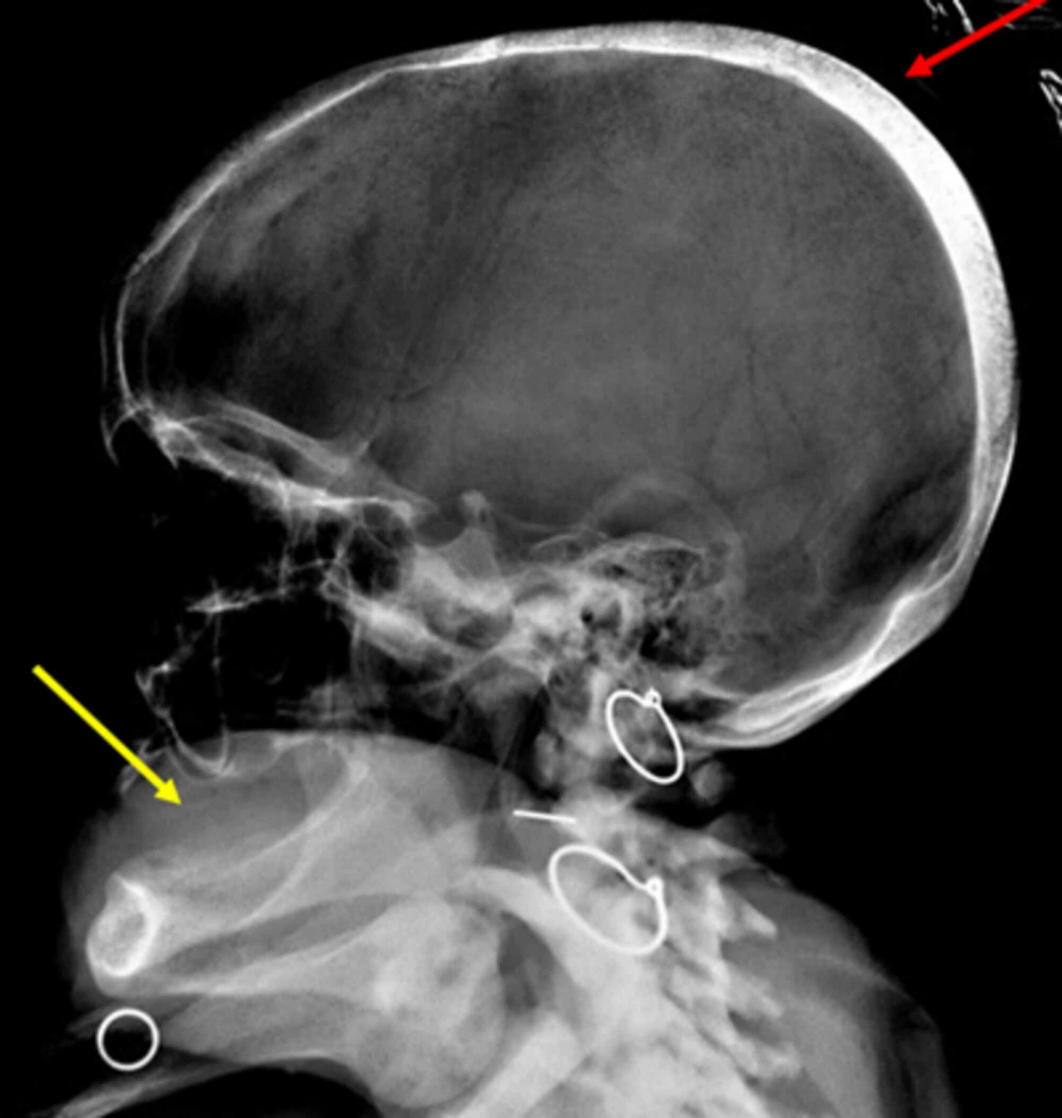
Skull radiograph The image shows cortical thickening at parieto-occipital calvaria (red arrow) and edentulous mandible, resorption of lamina dura with cortical thinning (yellow arrow)

Her pelvic radiograph revealed protrusio acetabuli and prominent bony spurs along tendon insertions suggestive of osteosclerosis and enthesopathy (Figure 1A). This was co-existent with osteomalacia of bilateral tibia and fibulae with insufficiency fracture at left tibial diaphysis (Figure 1B). Her spinal radiograph revealed bridging osteophytic changes and dorsolumbar scoliosis (Figure 2A). A calcaneal radiograph revealed calcaneal spurs, suggestive of degenerative and osteosclerotic changes (Figure 2B), although frank ossification of the Achilles tendon was not observed. Skull radiograph showed cortical thickening at parieto-occipital calvaria and edentulous mandible, with loss of lamina dura (Figure 3).

Given the early onset of FGF23-dependent hypophosphatemic rachitic disease, a possibility of XLH rickets was considered. Accordingly, a full gene sequence analysis of the PHEX gene was performed, which revealed a missense mutation of the PHEX gene.

The patient was started on calcitriol 0.25 ug twice daily and phosphate supplement at a dose of 30 mg/kg body weight/day in three divided doses. At the time of this write-up, the patient had completed one year of follow-up with us after the initiation of treatment. She has reported significant improvement in proximal myopathy along with the improvement of truncal weakness. Neurological examination revealed a power of 4/5 in the proximal and truncal muscles. There was also a significant decline in serum ALP to 634 IU/L (normal range: 80-240 IU/L), indicating improvement of metabolic bone disease. However, she did not have any significant improvement of the stiffness and pain around the joints. There was also not much improvement in her low backache.

## Discussion

XLH rickets is a genetic disease caused due to the mutation of the PHEX gene located on the X chromosome. The PHEX gene encodes for an endopeptidase that is responsible for the cleavage of FGF23. In the presence of PHEX mutation, FGF23 levels are elevated, which causes downregulation of NaPi-IIa and NaPi-IIc proteins, which are phosphate transporters located in the renal proximal tubules. As a result, there is excessive renal phosphate wastage, leading to hypophosphatemia.

Adequate amounts of serum phosphate are required for mineralization of the cartilage growth plate. The chondrocytes at the cartilage plate remain hypertrophied without undergoing apoptosis, giving rise to the expansion of the growth plates [6]. This leads to rachitic deformities when the child starts walking or putting pressure on the long bones.

Elevated FGF23 impairs 1-alpha hydroxylase enzyme, which is responsible for the conversion of 25(OH)vitamin D to its active form, which is calcitriol or 1 alpha, 25-dihydroxyvitamin D. Hence, calcitriol levels are low, leading to impaired intestinal calcium absorption, although hypocalcemia is usually not seen due to concomitant rise in the parathyroid hormone that helps maintain serum calcium levels.

Hypophosphatemia also accounts for poor dental health. The delayed eruption is noted for deciduous and permanent teeth [7]. There is an enlargement of pulp chambers and pulp horns [8]. Enamel hypoplasia is also commonly noted [9]. In histological studies of the dentin, large tubular clefts or lacunae are seen extending up to the dento-enamel junctions [10]. There is also impairment of dentin mineralization. This facilitates the invasion of bacteria into the dental pulps, leading to spontaneous dental abscesses. Frequent dental abscesses lead to tooth decay, causing premature loss of teeth.

Although the traditional hallmark of XLH is osteomalacia (a mineralization defect) [1], heterotopic ossification of tendon insertion sites has also been reported previously [2-4]. Our patient had classical radiographic images of osteosclerosis, hyperostosis, and enthesopathy. Enthesopathy refers to the involvement of ligamentous insertions of tendons in any pathological process. The exact mechanism of enthesopathy in XLH is not known, yet in a few previous studies, histological examination of the affected entheses has revealed the expansion of mineralizing fibrocartilage [5]. In the studies involving mouse models of XLH (Hyp mouse), it was found that ectopic ossification in XLH was not due to the action of bone-forming osteoblasts, rather it represented an expansion of the mineralizing fibrocartilage [5]. In the animal studies, it was also found that the entheses fibrocartilage cells expressed fibroblast growth factor receptor 3 (FGFR3)/Klotho [5].

The enthesopathy is clinically significant because it may be painful, may limit the range of motion, and may cause new-onset stiffness around the joints [5]. The predominant sites involved are the knees, ankles, pelvis, and thoracic spine. There may also be ossifications of the Achilles tendon, patellar tendon, and calcaneal insertion sites. Enthesopathy occurs late in the course of the disease and is usually resistant to treatment with phosphate or calcitriol. Hence, pain and stiffness may persist even after the resolution of other symptoms and the improvement of biochemical parameters.

## Conclusions

We described a case of a patient with XLH rickets who had rachitic manifestations and presented to us at 23 years of age with pain and stiffness around the joints. She was found to have extensive enthesopathy and hyperostosis. Although XLH is traditionally a defect of mineralization, heterotopic ossification and enthesopathy may also occur late in the course of the disease and may prove to be resistant to treatment.
